# Best Practice for De-Vulcanization of Waste Passenger Car Tire Rubber Granulate Using 2-2^′^-dibenzamidodiphenyldisulfide as De-Vulcanization Agent in a Twin-Screw Extruder

**DOI:** 10.3390/polym13071139

**Published:** 2021-04-02

**Authors:** Hans van Hoek, Jacques Noordermeer, Geert Heideman, Anke Blume, Wilma Dierkes

**Affiliations:** 1 Elastomer Technology and Engineering (ETE), Department of Solids, Surfaces and Systems (MS3), University of Twente, 7522 NB Enschede, The Netherlands; j.w.vanhoek@utwente.nl (H.v.H.); j.w.m.noordermeer@utwente.nl (J.N.); a.blume@utwente.nl (A.B.); 2 Professorship for Polymer Engineering, University of Applied Sciences Windesheim, 8017 CA Zwolle, The Netherlands; g.heideman@windesheim.nl

**Keywords:** de-vulcanization, recycling, passenger car tire, sustainability, twin-screw extruder

## Abstract

De-vulcanization of rubber has been shown to be a viable process to reuse this valuable material. The purpose of the de-vulcanization is to release the crosslinked nature of the highly elastic tire rubber granulate. For present day passenger car tires containing the synthetic rubbers Styrene-Butadiene Rubber (SBR) and Butadiene Rubber (BR) and a high amount of silica as reinforcing filler, producing high quality devulcanizate is a major challenge. In previous research a thermo-chemical mechanical approach was developed, using a twin-screw extruder and diphenyldisulfide (DPDS) as de-vulcanization agent.The screw configuration was designed for low shear in order to protect the polymers from chain scission, or uncontrolled spontaneuous recombination which is the largest problem involved in de-vulcanization of passenger car tire rubber. Because of disadvantages of DPDS for commercial use, 2-2${}^{\prime }$-dibenzamidodiphenyldisulfide (DBD) was used in the present study. Due to its high melting point of 140 °C the twin-screw extruder process needed to be redesigned. Subsequent milling of the devulcanizate at 60 °C with a narrow gap-width between the mill rolls greatly improved the quality of the devulcanizate in terms of coherence and tensile properties after renewed vulcanization. As the composition of passenger car tire granulate is very complex, the usefulness of the Horikx-Verbruggen analysis as optimization parameter for the de-vulcanization process was limited. Instead, stress-strain properties of re-vulcanized de-vulcanizates were used. The capacity of the twin-screw extruder was limited by the required residence time, implying a low screw speed. A best tensile strength of 8 MPa at a strain at break of 160% of the unblended renewed vulcanizate was found under optimal conditions.

## 1. Introduction

Rubber in general, and passenger car tire rubber in particular, heavily resist material degradation and survive for a long time in the environment. Due to the number of 10${}^{9}$ tires/year produced worldwide (derived from [1,2,3]), wasted passenger car tires pose enormous environmental problems when dumped on a land-fill, because of their intrinsic resistance against decomposition, or when being burned because of the soot and fumes they produce. These problems are addressed by industry and academia by developing processes for re-plastcization like reclaiming, and for decomposition like pyrolysis, with the aim to reuse the valuable resulting materials for new products. Reclaiming, a re-plasticization method, aims at breaking the polymer network in the tire rubber, created by the vulcanization process to obtain elastic rubber properties. Due to the high shear forces and processing temperatures applied in conventional reclaiming processes, not only the intended scission of cross-links occurs, but to a large extent also random scission of polymer chains. Although the rubber is plasticized, reclaimed rubber cannot readily be reused for high quality products due to the incurred degradation of the polymers. Furthermore, partial decomposition, chain scission and recombination of the polymer chains takes place, as is described by Golub [4] for thermal influences. Especially butadiene moieties contained in synthetic Styrene-Butadiene Rubber (SBR) and Butadiene Rubber (BR) are very susceptible to these kind of chemical reactions [5]. This happens over radical reaction paths during the reclaiming, triggered by interaction with atmospheric oxygen [6]. SBR and BR are predominantly used in passenger car tire compounds, much more so than Natural Rubber (cis-1,4-polyisoprene) used more commonly in truck tire compounds, where the latter is less prone to recombination and therefore much easier to de-vulcanize [7]. Markl and Lackner [8] discuss in their review paper the actual status of de- and revulcanization of car tires. However, the specific issues regarding de- and re-vulcanization of granulated passenger car tires, containing SBR and BR as synthetic polymers and a relatively high amount of silica as active filler, compared with carbon black, are not addressed.

Saiwari et al. [9] developed a thermochemical-mechanical de-vulcanization batch process on laboratory scale internal mixer for passenger car whole tire material using diphenyldisulfide (DPDS) as de-vulcanization agent, with treated distillate aromatic extract (TDAE) as process oil and tris(2,4-di-tert-butylphenyl)phosphite (TDTBP) as stabilizer. The use of a twin-screw extruder for de-vulcanization/reclaiming as a continuous operation has been researched before, Sutanto [10] through modelling a screw for the use of EPDM, using kneading elements to achieve a desired residence time, and by Saiwari [11] using this screw for de-vulcanization of ground tire rubber (GTR). A first attempt to scale-up a batch process for passenger car whole tire material to a twin-screw extruder continuous operation was reported by Saiwari et al. [12], making use of screws with nearly only conveying elements with flights of 1 D and 1.25 D. In order to maintain a residence time of 6 minutes (min), a low screw speed was used of 10 revolutions per minute (rpm). The so-called Horikx-Verbruggen analysis was employed for optimization from the perspective of retaining polymer integrity [13]. The optimal de-vulcanization formulation obtained was 18 mmol DPDS per 100 g of ground tire rubber (GTR), 6.2 wt% TDAE oil and 1 wt% TDTBP. Prior to feeding into the inlet of the extruder, the GTR was swollen with a mixture of the DPDS, TDTBP and processing oil. With this pre-treatment, kneading in the feed section of the extruder was not necessary.

A complicating factor is the influence of silica originating from the relatively recent silica-silane-based tread formulations used for passenger car tires, on de-vulcanization of GTR as well as on the morphology and tensile strength of the re-vulcanizates, was described earlier [14]. Approximately 23 phr (parts per hundred rubber) silica originating from the tire treads does indeed influence the vulcanization kinetics of the de-vulcanizates as well as the tensile properties of the corresponding renewed vulcanizates. This issue is integrated in the present study. Another complicating factor was the insufficient commercial availability of DPDS for large-scale operations, so another de-vulcanization agent had to be selected. With 2-2${}^{\prime }$-dibenzamidodiphenyldisulfide (DBD) as a higher-melting de-vulcanization alternative, the screw design had to be adjusted, compared with the one used by Saiwari et al. [12]. By pre-mixing in an available mixer the DBD accumulated at the mixer wall and in other locations, hence manual pre-mixing was applied before feeding the mixture into the hopper. The de-vulcanization agent had now to be molten and thoroughly homogenized with the GTR in the first mixing section of the extruder to allow the DBD to migrate into the granulate particles. Because additional screw length was needed for this mixing section, gravity feeding was chosen as the whole screw length can then be used.

An important question was how severe a kneading was allowed for sufficient homogenization, while keeping the motor power at an acceptable level for the non-plasticising rubber material. This is one important point of this study. All these complicating factors make de-vulcanization/reclaiming of largely synthetic rubber based whole passenger car tires considerably more difficult than for NR based truck tire material. The purpose of the present study was therefore to identify solutions and develop optimal conditions for continuous twin-screw extruder de-vulcanization of state-of-the-art passenger car whole tire material, while keeping the polymers, mainly SBR, as much as possible intact. So as to obtain maximally attainable properties for second use.

## 2. Theoretical Considerations Concerning Distribution and Diffusion of De-Vulcanization Agents into Rubber Granulate

The fact that DBD as de-vulcanization agent cannot be swollen into the rubber at ambient temperature has two consequences:

1. The extruder configuration needs to provide thorough mechanical mixing of the GTR with the DBD;
2. The operating extruder temperature has to be on a level that DBD melts and can migrate into the GTR particles.

These elements of physical transport pose practical limitations on the process. Using tire granulate with a typical particle diameter of 1 mm to 3.5 mm, the following points have to be considered:

- The concentration of de-vulcanization agent related to the volume of the particles depends on the surface area to volume ratio of the granulate. The de-vulcanization agent is distributed over the surface of the particles during mixing, before it can migrate into the particles;
- A certain time is required for the process oil and de-vulcanization agent to migrate into the granulate particles;
- Before equilibrium within the particles is reached, a time-dependent concentration gradient from the surface to the center exists;
- Similarly, a time-dependent temperature gradient is occurring.

### 2.1. Concentration of the De-Vulcanization Agent Depending on the Particle Size Distribution of the Granulate

Assuming spherical particles, the difference in surface to volume ratio between the largest, ~3.5 mm, and the smallest, ~1 mm, particles is a factor of 3.5. The effect of surface roughness is neglected. When mixing the GTR with the process oil and de-vulcanization agent, these components will initially be distributed over the surface of the granulate particles. Due to the higher volume to surface ratio of the larger particles compared to the smaller ones, only one third of the oil and de-vulcanization agent will deposit on the largest granulate particles compared to the smallest ones. Consequently, a disproportionally high amount of oil and de-vulcanization agent settles on the smaller particles due to the relatively high number of such smaller granulate particles in the GTR. Based on the size distribution of the GTR as measured by sieving—particles with a diameter of 2 mm to 3.5 mm formed the largest weight fraction of about 80 wt% of the sample, and the fraction with a diameter of 0.85 mm to 2 mm amounted to about 20 wt%. A small fraction of 1 wt% of particles larger than 3.5 mm was found [12]. For the GTR, to which an overall amount of process oil of 5 wt% was added, therefore about 9 wt% of oil was absorbed by the smallest fraction versus 2 wt% by the largest one, when using cut-offs of 0.85 mm, 2 mm and 3.5 mm. Similar differences in concentration of the de-vulcanization agent apply. The overall conclusion from this consideration is that after migration of the oil and de-vulcanization agent into the particles, the largest ones will experience a considerably lower concentration of additives than the smallest, resulting in an inherent inhomogeneity on the particles scale.

### 2.2. Concentration Gradient of the De-Vulcanization Agent inside a Particle

When mixing the de-vulcanization agent with granulate, it needs to migrate into the particles in order to achieve an equilibrium concentration. For a model description of this situation a constant concentration of a liquid outside a particle may be assumed [15]. In actual practice, the amount of liquid on the surface will decrease while it diffuses into the particle. Based on the fact that de-vulcanization agent and oil must migrate to the core of the particles, it takes much longer before the concentration in the center reaches a level needed to initiate de-vulcanization for a large particle than for a smaller one. Bouvier and Gelus [16] investigated the migration of heavy oil into SBR as a function of temperature. Although the concentration of oil outside the particle was kept constant, their results can be used for a first estimation of the time scale of the migration using Fick’s law. For the system oil-SBR, Bouvier measured the diffusion coefficient D as a function of temperature and defined the diffusion time $t_{D}$ to equilibrium as:

(1) $$t_{D}=R^{2}/D,$$

where *R* presents the radius of the equivalent sphere.

With Equation (1) and initial diameters of the particles of 2 mm and 3 mm, an estimation of the time needed for migration of the process oil into the particles till equilibrium can be made, see Table 1.

At 200 °C, the process temperature of the de-vulcanization experiments (see Section 4.3), the diffusion time till equilibrium for the larger particles is of the same order as the residence time in the extruder, about 6 min. At 163 °C, still above the melting temperature of DBD 140 °C, the diffusion time increases to 22 min: too long to be feasible. Hence, although it is only an estimation, the migration times for large particles are significantly longer than the available residence time in the extruder.

### 2.3. Temperature Gradient inside a Granulate Particle

The temperature gradient inside a sphere as a function of time follows a similar relation as the concentration gradient [15]. An estimation can be made for the required time for the center of a particle to reach a certain temperature, assuming spherical shape of the particles. To reach 200 °C throughout the entire granulate, and with an initial temperature of 30 °C and temperature setting of the extruder at 220 °C, then:

(2) $$\frac{T-T_{0}}{T_{1}-T_{0}}=0.89,$$

with *T*${}_{0}$ the initial temperature of the granulate, *T*${}_{1}$ the temperature at the surface for t > 0 and *T* the temperature at the core. Based on the master curves presented in [15], 0.89 correlates to a value of $\alpha t/R^{2}\approx 0.35$, were α = the heat transfer coefficient, t = time and R = radius of the sphere. For SBR, a major component of the granulate composition, it can be derived [17] that:

α=κ/ρc = $1.06\times 10^{-7}$ m^2^/s,
with κ = thermal conductivity = 0.2 W/m K,
ρ = density = 940 m^3^,
c = specific heat capacity = 2 kJ/kgK.

For the larger particles with a diameter (=2R) of 3 mm, the time needed until the core reaches 200 °C at an extruder setting of 220 °C, can be derived as [15]:

(3) $$\frac{\alpha t}{R^{2}}=0.35⟶t\approx 7.5\;s.$$

This shows that heat is transported quickly throughout the particles and can therefore be considered to be of minor influence on the inhomogeneity of the de-vulcanization of the various particles.

## 3. Materials and Methods

### 3.1. Materials

The GTR used in this investigation was obtained from Genan, Dorsten, Germany. It is a commercial ground passenger car tire granulate, medium grade, containing at least 45 wt% polymer of which 10 wt% to 35 wt% Natural Rubber (NR) and an ash content (mainly silica) of less than 10 wt%. The particle sizes range from 1 mm to 3.5 mm [18]. Using a set of laboratory sieves, the size distribution as presented in Table 2 was obtained. This GTR type was selected based on its low level of contaminations originating from stones or dirt adhering to the tire-surface before granulating, steel from the reinforcing cords in the beads of the tires or wear of the cutting and grinding equipment, and dust from fibers in the tire carcass. The coarser grades usually contain higher levels of dust, steel and fibers from the tire carcass, the finer grades usually contain higher amounts of steel and stone dust.

The de-vulcanization agent 2-2${}^{\prime }$-dibenzamidodiphenyldisulfide (DBD) used for the present study is a commonly used peptizer or mastication agent for Natural Rubber. DBD melts at 140 °C, therefore pre-swelling of the tire granulate with a blend of molten DBD and process oil is not possible as the rubber will start to degrade when kept at a temperature above 140 °C for the duration of the swelling process. Manual mixing of GTR with processing oil, DBD and TDTBP prior to feeding to the extruder inlet was therefore opted for. This implied thorough mixing of the GTR during melting of the DBD in the mixing section of the extruder.

The origins of all materials employed in the present study are: 2-2${}^{\prime }$- dibenzamidodiphenyldisulfide (DBD) from Schill and Seilacher GmbH, Boeblingen, Germany. Tris(2,4-di-tert-butylphenyl)phosphite (TDTBP) from Sigma Aldrich Cooperation, Zwijndrecht, The Netherlands. Treated Distillate Aromatic Extract (TDAE), VIVATEC 500 process oil from Hansen & Rosenthal, Hamburg, Germany. Acetone, purity > 99.5 wt%, tetrahydrofuran (THF), purity > 99.8 wt% and toluene, purity > 99.8 wt% were all purchased from Atlas & Assink Chemie b.v., Enschede, The Netherlands. Butadiene Rubber (BR) grade BUNA CB24 was from Arlanxeo Deutschland GmbH, Leverkusen, Germany. TiO_2_ (titanium dioxide), Hombitan R210 from Venator, Wynyard, UK. ZnO (zinc oxide) and stearic acid from Merck KGaA, Darmstadt, Germany. Sulfur from J.T.Baker. N-tert-butyl-2-benzothiazolesulfenamide (TBBS), mercapto benzothiazoledisulfide (MBTS) and 1,3-diphenylguanidine (DPG) were all from Lanxess Rhein Chemie GmbH, Cologne, Germany, and bis[3-(triethoxysilyl)propyl] tetrasulfide (TESPT) from Evonik Industries AG, Essen, Germany. NaOH, technical quality, from Sigma Aldrich Cooperation, Zwijndrecht, The Netherlands. Bleaching water, a 2% solution of NaHClO in water.

### 3.2. Quality Analysis Methods for De-Vulcanizates

In this section the quality analysis methods as used for this investigation are discussed: Cure characteristics, tensile tests, the Horikx-Verbruggen analysis and the white rubber analysis.

#### 3.2.1. Cure Characteristics and Compression Molding

Compounds based on de-vulcanized material were tested for their cure characteristics with a Rubber Process Analyzer, RPA Elite from TA Instruments, at 170 °C, 0.833 Hz, and 2.89% strain according to ISO 6502. For renewed vulcanization of the de-vulcanizates a Wickert WLP1600 laboratory compression molding press was used at 170 °C for a period of tc_90_ + 2 min, using a mold of 100 mm × 100 mm × 2 mm.

#### 3.2.2. Tensile Tests

Tensile testing was done with a Zwick BZ1.0/TH1S tensile tester using dumbbell shaped samples according to ISO 37 type II with a crosshead speed of 500 mm/min. The tensile strength and strain at break of the re-vulcanizates were taken as optimization criteria, as from an application perspective these are properties of prime importance—without at least reasonable tensile properties, the re-vulcanizates will hardly be usable for any application. On the other hand, in actual applications it is not likely that pure de-vulcanizates will be used but rather as a blend with virgin elastomers. So this criterion is very severe.

#### 3.2.3. Horikx-Verbruggen Analysis

**Figure 1:**
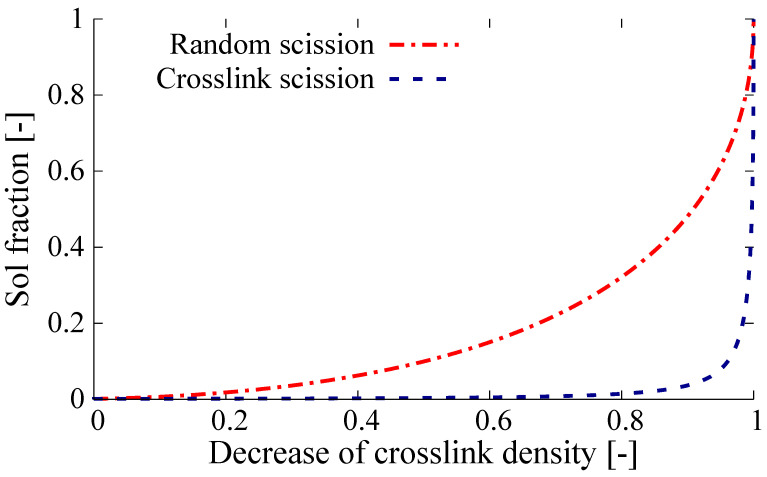
A typical Horikx-Verbruggen diagram.

In order to quantify the amount of random polymer scission versus cross-link scission, the method of Verbruggen [13] was employed, based on the original Horikx [19] theory for polymer network breakdown by highly energetic radiation. In this method the fraction of polymer detached from the network and thus soluble is plotted against the relative decrease in the cross-link density. This allows to differentiate between unwanted random scission of the polymer and preferred cross-link scission, see Figure 1.

For this purpose, the network densities are commonly measured by means of the Flory-Rehner swelling approach [20], which was originally developed for non filler-reinforced polymer networks. Kraus [21] and Porter [22] have shown that this method is also applicable for carbon black filled rubber. They applied a correction factor for the amount of filler. Verbruggen et al. demonstrated that the method can also be applied to more complex single polymer networks. The soluble sol-fraction was determined by extraction of the samples with acetone and subsequently with THF, using a Soxhlet apparatus with drying and weighing steps before and after extraction. After swelling the extracted samples in toluene, the cross-link density could be calculated from the amount of absorbed liquid, using the theory of Flory-Rehner and applying the correction factor derived by Porter.

#### 3.2.4. White-Rubber Analysis

A White Rubber Analysis (WRA) was employed for the present purpose in order to quantify the number of irregularities (visible grains) in the de-vulcanizates, as these have an influence on miscibility and renewed vulcanizate properties. A blend of 95 wt% of a BR-based compound with 65 phr titanium dioxide and 5 wt% de-vulcanizate provided the best visibility of the grains on a grey background over the whole range of produced de-vulcanizates. The blends were vulcanized in a mold to a circular disk of 5 mm with a diameter of 50 mm. After sanding to remove the surface layer until a homogeneous distribution is visible, the samples showed a gray surface with embedded black spots, corresponding to the number and size of the grains in the de-vulcanizates. Pictures were converted to black and white to improve the contrast. Although a statistical analysis of the characterizations was developed, a visual comparison appeared most illustrative and was applied in the present study.

### 3.3. Equipment and Experimental Set-Up for De-Vulcanization and Re-Vulcanization

As pre-treatment of the GTR it was mixed with process oil, DBD and stabilizer was performed manually for a maximum batch size of 7 L in a simple container, as the powdery DBD stuck to the wall of the mixer previously used for mixing and swelling of DPDS.

For compounding of the de-vulcanizate for re-vulcanization, a Brabender Plasti-corder internal mixer with a chamber volume of 50 mL was used.A Schwabenthan laboratory mill with rolls of 200 mm width, a roll diameter of 80 mm and a friction ratio of 1.13 was used at 22 rpm, 40 °C to 60 °C and a gap width between the rolls of 0.1 mm to 2 mm for the final milling of the de-vulcanizate after the calender and for all milling after renewed mixing. A complicating factor in the qualitative analysis of the de-vulcanizates by tensile testing of re-vulcanisates is that, beforehand, it is not known whether and how much silica is contained in commercial samples, as opposed to sole use of carbon black as reinforcing filler. The pertinent compounding formulations needed are fundamentally different and lead to different results. For that reason a double approach was taken:

- Analysis of the de-vulcanizates with a carbon black based compound formulation for re-vulcanization;
- Analysis of the de-vulcanizates with a formulation tuned to a mix of CB and silica as reinforcing fillers.

The corresponding compound re-vulcanization formulations and compounding procedures are given in Appendix A.

**Figure 2:**
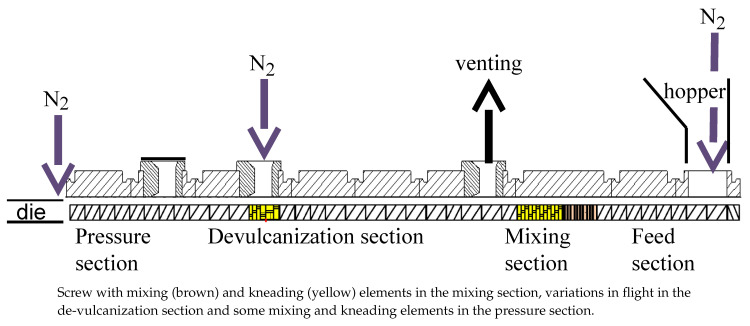
Layout of the extruder, from right to left, with an example of a screw with a limited amount of kneading and mixing elements after the mixing section, and nitrogen supply positions.

**Figure 3:**
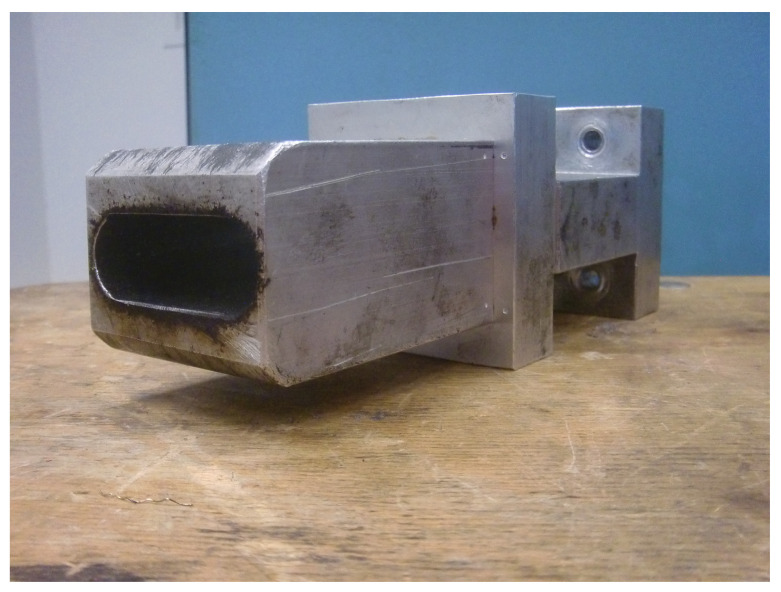
Elongated die.

**Figure 4:**
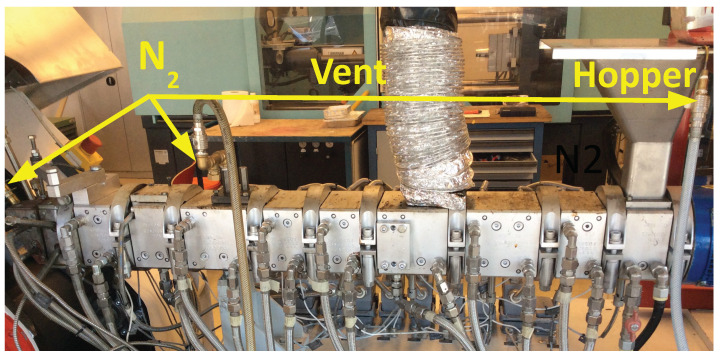
Extruder with the supply hopper, ventilation point and nitrogen supplies.

**Figure 5:**
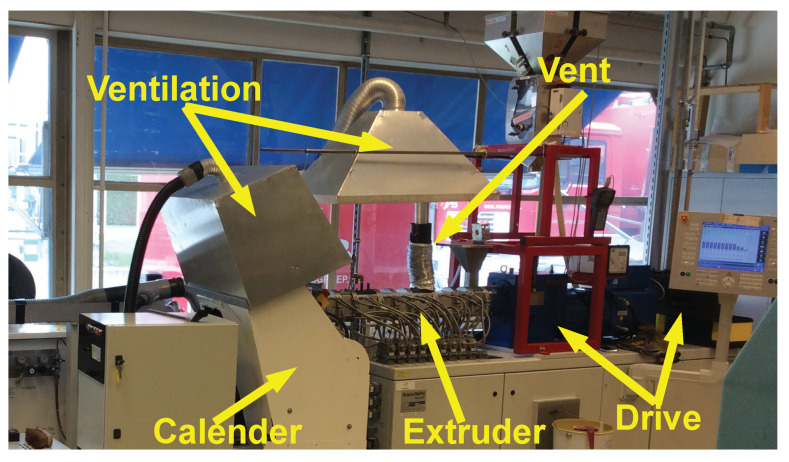
Extruder with calendar and venting system.

The overall setup of the de-vulcanization installation is shown in Figure 2, Figure 3, Figure 4 and Figure 5. The continuous de-vulcanization was performed using a KrausMaffei ZE 25 UTX co-rotating twin-screw extruder (KraussMaffei Technologies GmbH, München, Germany), with a length of 42D with D = 25 mm, and equipped with 3 de-gassing positions between the inlet and outlet, of which one is used for nitrogen supply and one for venting, as shown in Figure 2. Contrary to thermoplastic polymers wherefore a twin-screw extruder is commonly employed to melt and subsequently mix the materials, in the present context the rubber crumb does not melt. The purpose of the extruder is to “knead the de-vulcanization aid into the granulate” so as to selectively release crosslinks in a controlled manner, in order to at the end have material in a re-plasticized state. The rubber remains in granular form throughout the process of de-vulcanization. This is contrary to reclaiming, wherein from the onset already small polymer fragments are broken off the network and behave as re-plasticized material. It requires re-thinking of the basic concepts of twin-screw extruders. The effects of kneading and mixing elements differ considerably. Negative flight zones meant for pressure build-up tend to obstruct the flow of the granulate in the extruder. They create a three-dimensional load situation which the granulate absorbs by its elastic nature, being compressed against the barrel wall without transport, generating increased friction instead of a two-dimensional load situation in a thermoplastic melt, where in the latter case the pressure increase is limited by the flow of the melt. This has strong implications for understanding the behavior of the granulate in the extruder during the de-vulcanization process as well as for the selection of a proper screw configuration. This is therefore an important part of the present study. For similar reasons, the twin-screw extruder was not run in the so-called starve-fed mode, as is more common for thermoplastic polymers. Above the extruder inlet a hopper was mounted for continuous dosing of the whole tire granulate. An elongated die of rectangular shape, 20 mm × 40 mm and length of 100 mm, with rounded edges was mounted at the extruder outlet to increase the residence time, as shown in Figure 3.

The extruder was operated at a screw speed of 10 rpm to 30 rpm. At 20 rpm, the overall residence time of the de-vulcanizate in combination of the extruder and elongated die was approximate 12 min. At this speed, the pressure in the extruder before material enters the die reached a maximum of 5.4 MPa, depending on the temperature settings of 180 °C to 220 °C in the de-vulcanization section. The pressure before the die varied between 1.9 MPa to 5.4 MPa and the load indication of the extruder drive between 19% to 66%. A direct correlation of these parameters with the added concentration of oil or the concentration of DBD was not observed.

In order to minimize oxidative degradation during de-vulcanization, the extruder was equipped with nitrogen supply—in the hopper, at the end of the de-vulcanization section and at the beginning of the elongated die, see Figure 2 and Figure 4. The de-vulcanizate was transferred to a specially constructed cooling calender [23], positioned directly directly after the extruder, see Figure 5. This calendar cooled the de-vulcanizate very quickly and efficiently to 40 °C to 60 °C to prevent oxidation, it was not designed to apply high pressures or shear forces to the devulcanized material. The temperature of the cooling water was controlled to prevent condensation on the calender rolls. The capacity of this total line is approximate 2 kg/h at a screw speed of 20 rpm.

**Figure 6:**
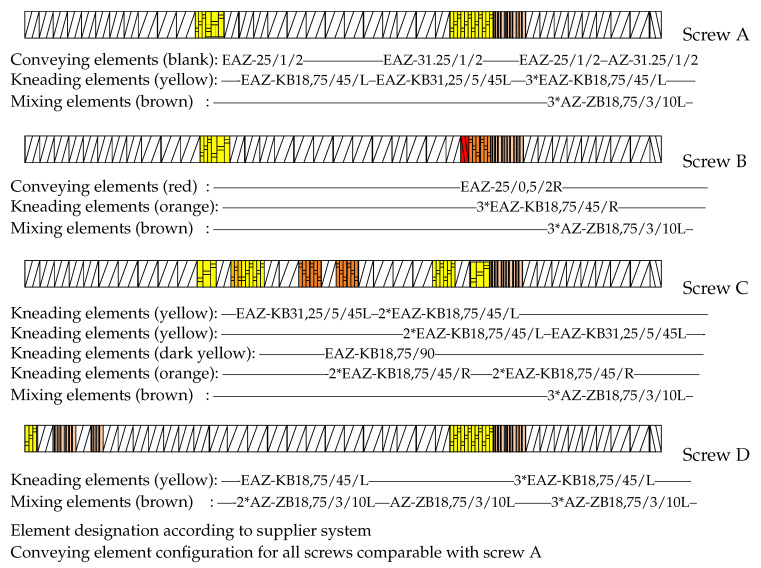
Screw configurations with different amounts of shear adapted for use with dibenzamidodiphenyldisulfide (DBD).

With the use of DBD as de-vulcanization agent: the de-vulcanization agent had to be molten and thoroughly mixed with the GTR in the mixing section of the extruder, see Figure 2. Kneading in this section was required and subsequently the shear in the de-vulcanization section was adjusted. Screw configuration A, Figure 6, was chosen so as to increase the shear in the mixing section as a first modification. Configuration B was chosen to extend the residence time in the mixing section of the screws to prolong the swelling time. Configuration C included more kneading elements in the mixing section for more thorough mixing and some additional right and left hand kneading elements in the de-vulcanization section. Configuration D was chosen as a variation on configuration A, with additional shear in the pressure section of the extruder by adding some mixing and kneading elements. After testing, it appeared that for screw setups B and C, the drive could not provide the required high torque due to the additional friction caused by the right-handed elements. Screws A and D were finally selected for further testing.

## 4. Results and Discussion

### 4.1. De-Vulcanization Parameters

To optimize the extruder parameters for de-vulcanization, the following settings were considered:

- Screw configuration: Two screw configurations were selected for further testing: A and D.
- Extruder temperature profile: Three sections of the extruder can be distinguished, and for each section the optimal temperature had to be selected, see Figure 2: The feed- and mixing section:
  The heating of the GTR and the DBD so that optimal migration of the DBD into the GTR is achieved, but prevents the start of the de-vulcanization to avoid breakdown of the polymer due to the initial high concentration of the DBD. A selection was made out of 100 °C, 130 °C and 220 °C.
  The de-vulcanization section: Setting of the optimal de-vulcanization temperature. A selection was made out of 180 °C, 220 °C, 230 °C and 240 °C.
  The pressure section: Adjust the temperature setting to prevent too high temperatures due to pressure development on the de-vulcanizate. A selection was made of 100 °C, 120 °C, 150 °C, 170 °C, 190 °C and 220 °C.
- Most important is the influence on the residence time as the result of varied screw-speed. A selection was made of 10 rpm, 17 rpm, 20 rpm, 30 rpm and 34 rpm.
- Amount of DBD: To find the minimum concentration necessary for de-vulcanization a selection was made of 3.9 wt%, 5 wt% and 6.85 wt% DBD relative to ground rubber, derived from the amounts of DPDS as used by Saiwari [12].
- Concentration of processing oil: The processing oil is used to soften the GTR in order to enhance the migration of the de-vulcanization agent into the granulate. As undesired side effect, it decreases the shear during de-vulcanization. A selection was made of 0 wt%, 2 wt%, 5 wt% and 6.2 wt% oil relative to ground rubber.
- For the stabilizer TDTBP, a concentration of 1 wt% relative to ground rubber was used.

### 4.2. Distributive Mixing of De-Vulcanization Agent and Granulate Particles in the Extruder

As discussed in Section 2.1 and Section 2.2, the concentration of de-vulcanization agent on the surface of the granulate particles is expected to vary with particle size and time. To have an indication of transfer of de-vulcanization agent between the various smaller and larger particles, a small experiment was performed using DPDS as de-vulcanization agent: white vulcanized rubber as used for the WRA was granulated to a size of approximately 4 × 4 × 2 ${\mathrm{mm}}^{3}$. One part of this granulate was swollen with DPDS, while the other half was kept untreated. Both samples were separately fed into the extruder, with sufficient separation in time to allow all white rubber to be discharged. By visual observation, the untreated white rubber did not show any recognizable indication of de-vulcanization, while the treated sample clearly did. This indicates that the transfer of de-vulcanization agent between the granulate particles is rather limited, and stresses the importance of swelling of the DBD into the granulate either as pre-treatment or in the first section of the extruder.

### 4.3. Screening Optimization Criteria for De-Vulcanizate Quality

**Figure 7:**
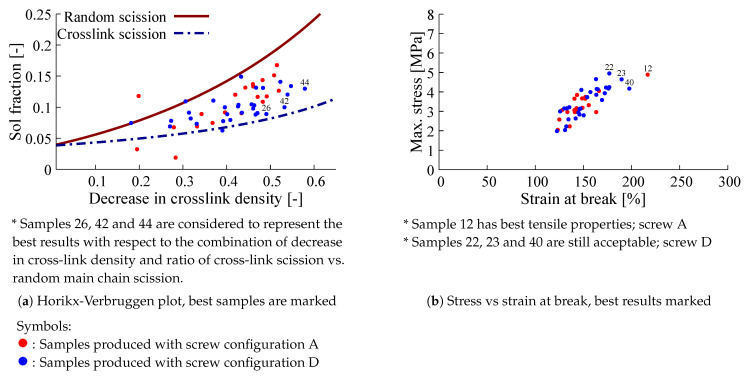
Preliminary de-vulcanization data presented as both, Horikx-Verbruggen points and stress-strain at break points after having been re-vulcanized using the CB -based formulation given in Table A1, Appendix A.1.

**Figure 8:**
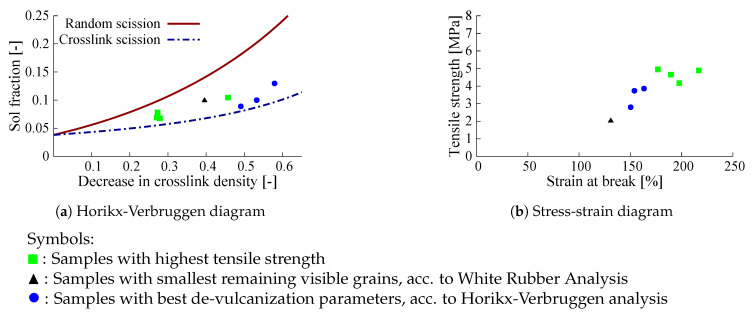
Samples with optimal decrease in cross-link density, smallest particle size and highest tensile strength in Horikx-Verbruggen cq. stress-strain diagram.

After preliminary de-vulcanization experiments of GTR with various extruder settings the obtained samples were analyzed using the Horikx-Verbruggen method as shown in Figure 7a. Subsequently, the de-vulcanizates were re-vulcanized using the CB-formulation and procedure as described in Section 3.3: Table A1 and Table A2 of Appendix A.1. The stress and strain at break after re-vulcanization are given in Figure 7b. From these data it can be concluded that screw configuration D gives better results in terms of the ratio of random to cross-link scission. With respect to stress- strain properties, the results are a bit inconclusive, as can be seen in Figure 7b: screw A results in one sample with the best performance, but the majority of samples processed wit screw D perform overall the best. The sample numbers in the figures are of no particular meaning in the present context. The best samples with respect to degree of de-vulcanization taken from the Horikx-Verbruggen diagram and tensile strength are highlighted in Figure 8: Unexpectedly, the degree of de-vulcanization according to the Horikx-Verbruggen analysis and tensile properties do not match—the samples with the highest degree of de-vulcanization (blue circular marks in Figure 8a) do not show the highest strength values after re-vulcanization, see Figure 8b, and vice versa. From this, it can be deduced that the position in the Horikx-Verbruggen diagram is not a suitable optimization parameter for practical purposes. Although the Horikx-Verbruggen analysis has shown to be reliable in previous cases [13], some remarks can be made concerning this apparent controversy:

- As stated before, SBR tends to re-cross-link by recombination of radicals during the de-vulcanization process. This changes the structure of the polymer, which has a negative impact on the overall result of the de-vulcanization and properties of the re-vulcanizate;
- Carbon black based NR compounds are known to de-cross-link relatively easily;
- Remaining hard particles, visible grains in the samples, assumed to be material de-vulcanized to a lower degree, offset the mean value of the cross-link density.

The apparent contradictory differences seen in Figure 7 and Figure 8 can also be due to the presence of silica in the GTR. As this GTR based on passenger car tires contained a considerable amount of silica-silane, the following additional remarks have to be made:

- To determine the cross-link density with the Flory-Rehner method [20], the degree of swelling must be corrected for the influence of fillers. As it is unlikely that the correction factor, as proposed by Porter [22], is similar for carbon black and silica-silane filler systems, the cross-link density calculation for a compound with both, carbon black and silica as fillers, is not fully reliable.
- Compounds containing silica as reinforcing filler are known to de-cross-link only to a low degree, mainly because of the high number of mono-sulfidic cross-links from the silica-silane-polymer bonds, which cannot selectively be broken in this process. This results in a certain inhomogeneity in the de-vulcanizates.

**Figure 9:**
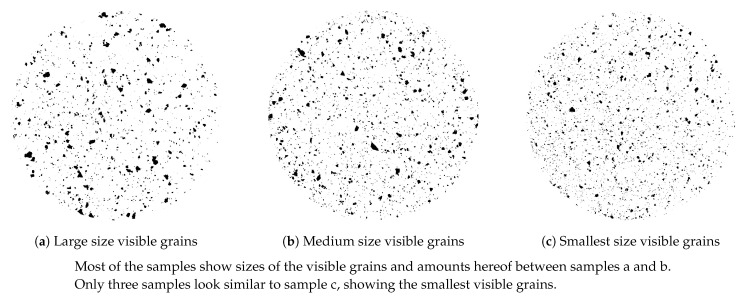
White Rubber Analysis of de-vulcanizates differing in particle size distribution; the diameter of the samples is 50 mm.

Size and amount of the visible grains remaining in the de-vulcanizates were determined using the WRA method. Most of the samples produced in this study showed particle size distributions comparable to sample (a) and (b) with mainly large- and medium size visible grains, as shown in Figure 9a,b. Only three samples showed rather small visible grains, see Figure 9c.

**Figure 10:**
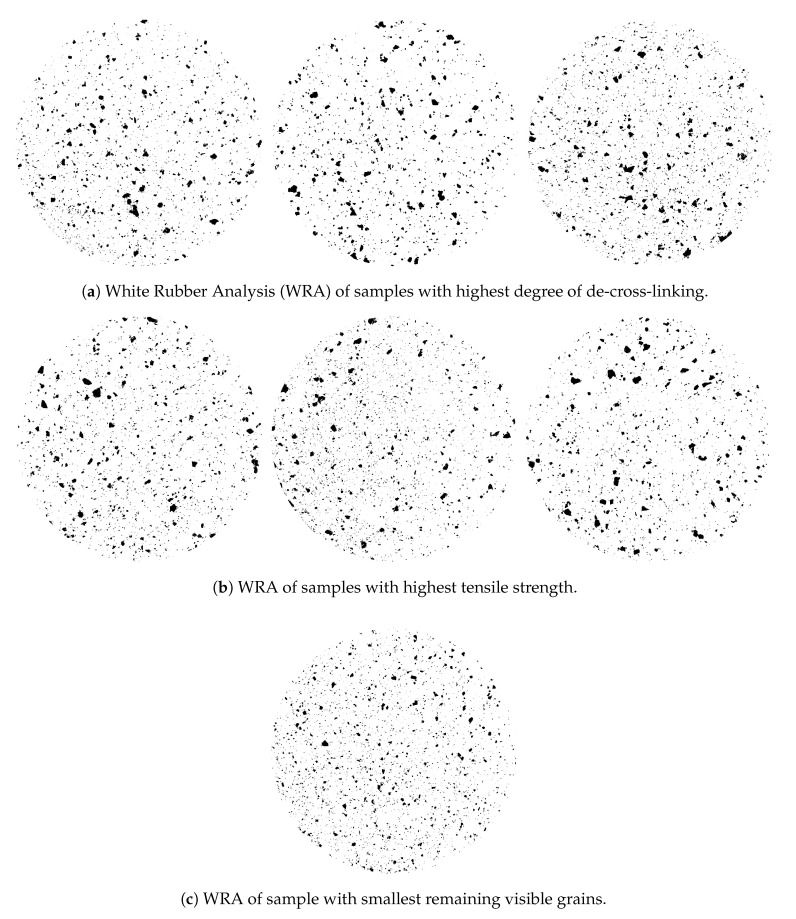
Comparison of remaining particle size distribution by WRA related to decrease in cross-link density and tensile strength. The diameter of the samples is 50 mm.

For all samples shown in Figure 8, the particle sizes as determined by WRA are shown in Figure 10. The particle sizes for the best samples with respect to de-vulcanization degree as indicated by the Horikx-Verbruggen analysis are shown in Figure 10a and those with best tensile strength in Figure 10b. The sample with the smallest particle size, Figure 10c, shows an intermediate de-vulcanization degree and the lowest tensile strength of all. Although the size and amount of the visible grains as seen in the WRA did not show a clear correlation with tensile strength and ultimate strain properties of the re-vulcanizates, it was anyway taken along as secondary parameter to optimize the processing after de-vulcanization, as the size of the remaining visible grains in the de- and re-vulcanizates will be seen as quality items. Based on these considerations, the conclusion must be that the Horikx-Verbruggen analysis is of limited value for this kind of inhomogeneous materials. Tensile strength of re-vulcanizates is a better choice for optimization of the de-vulcanization process. However, the presence of silica and its influence on the curing behavior during re-vulcanization has to be taken into account. Therefore, formulations I and II (Table A3 in Appendix A.2 ) adjusted for a silica based tread formulation using the processing steps described in Table A4, were further employed in the present study.

### 4.4. Optimization of the De-Vulcanization Process Parameters Based on Tensile Strength

**Figure 11:**
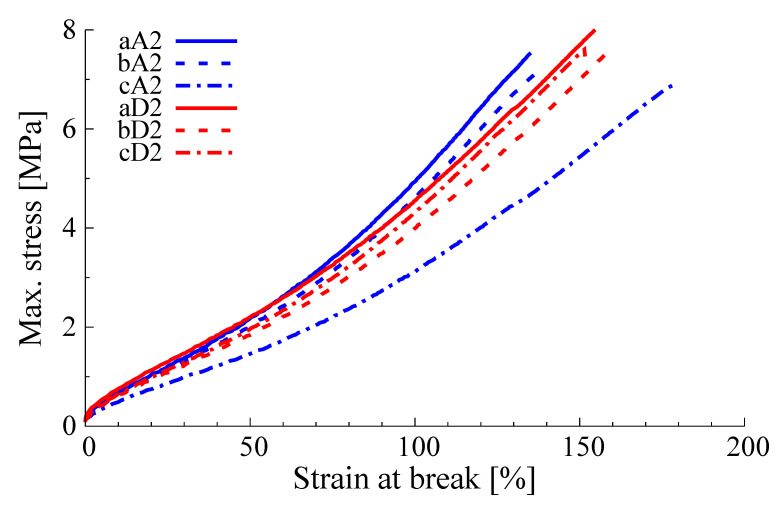
Stress-strain diagram of the three samples with highest tensile strength for screw configurations A and D. See Table 3 and Table 5.

A representative number of samples was used for further testing in order to adjust the formulation to the presence of silica. After re-vulcanization of the de-vulcanized samples using the silica-based formulation in Table A3, and procedure in Table A4, the tensile strength and strain at break were measured. Results from de-vulcanization tests using screw configuration A and re-vulcanized using silica formulation II as shown in Table A3, Appendix A.2 with an increased amount of curatives to overcome the low vulcanization activity due to the silica, are shown in Table 3. Results from using screw configuration D and re-vulcanized using formulation I in Table A3 are shown in Table 4, and using the same screw but formulation II in Table 5. The stress-strain curves for the samples with the best tensile strength for screw configurations A and D and re-vulcanized with formulation II are shown in Figure 11.

The following observations can be made concerning the various conditions explored:

**Temperature:** The samples that are devulcanized at max. 180 °C show a better tensile strength than those devulcanized at 220 °C;
**Amount of DBD:** There is no significant difference in tensile strength between the samples devulcanized with 3.9 wt% DBD and those with 6.85 wt%;
**Screw configuration:** The results as presented in Figure 7 indicate a better performance of screw D compared to screw A. This is also indicated in the data in Table 3, Table 4 and Table 5. These samples show higher tensile strength values at a screw speed of 20 rpm for screw D compared to 10 rpm for screw A, although here is also a difference in screw speed involved: see next.
**Screw speed:** Most of the samples with a good tensile strength are obtained at a screw speed of 20 rpm. The tensile strength of the sample bD2, Table 5, is also one of the highest, but produced with a screw speed of 30 rpm, 6.85 wt% of DBD combined with a de-vulcanization temperature of 220 °C. Apparently, a screw speed of 20 rpm combined with a devulcanization temperature of 180 °C shows a similar performance as a screw speed of 30 rpm combined with a devulcanization temperature of 220 °C. It indicates a possible trade-off between energy efficiency and throughput.
**Amount of oil:** All samples with the higher tensile strengths are produced using a low amount of 2 wt% oil.
**Re-vulcanization formulation:** As a secondary criterion, the strain at break can be used. This property is slightly better for the samples re-vulcanized with formulation II compared to formulation I. In both cases, screw B was used with 2 wt% of oil.

### 4.5. Summary of the Best De-Vulcanization Process Set-Up

**Figure 12:**
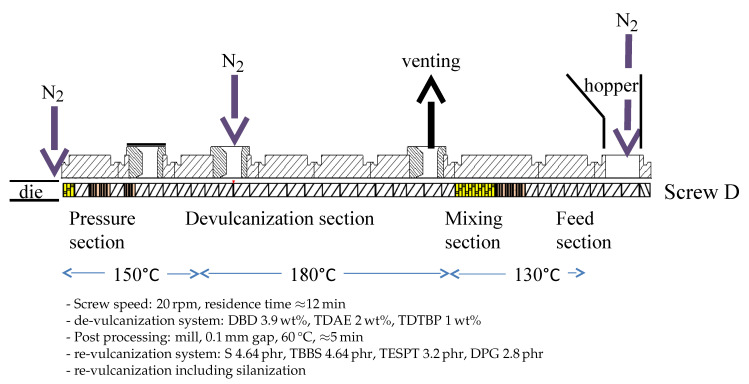
Extruder configuration and de-vulcanization process parameters for best tensile strength of re-vulcanizates.

These observations lead to the conclusion that a de-vulcanization temperature of 180 °C combined with screw configuration D and 2 wt% oil are the best settings for the de-vulcanization process. As there is no significant advantage of using 6.85 wt% DBD over 3.9 wt%, the latter suffices. Re-vulcanization using formulation II gives slightly better strains at break. This is all summarized in Figure 12.

### 4.6. Benefits of Post-Processing Step

The de-vulcanization process as developed in the present study is a thermo-chemical-mechanical treatment. The shear created by the kneading and mixing elements in the de-vulcanization and pressure sections of the screws is not sufficient to fully decrease the amount and size of the visible grains in the de-vulcanizates. Screw D, configured for higher shear in the pressure section, did not improve this contrary to expectations. Additional post-process milling of the DGTR at 60 °C, with a gap width of 0.1 mm had a clear positive effect.

**Figure 13:**
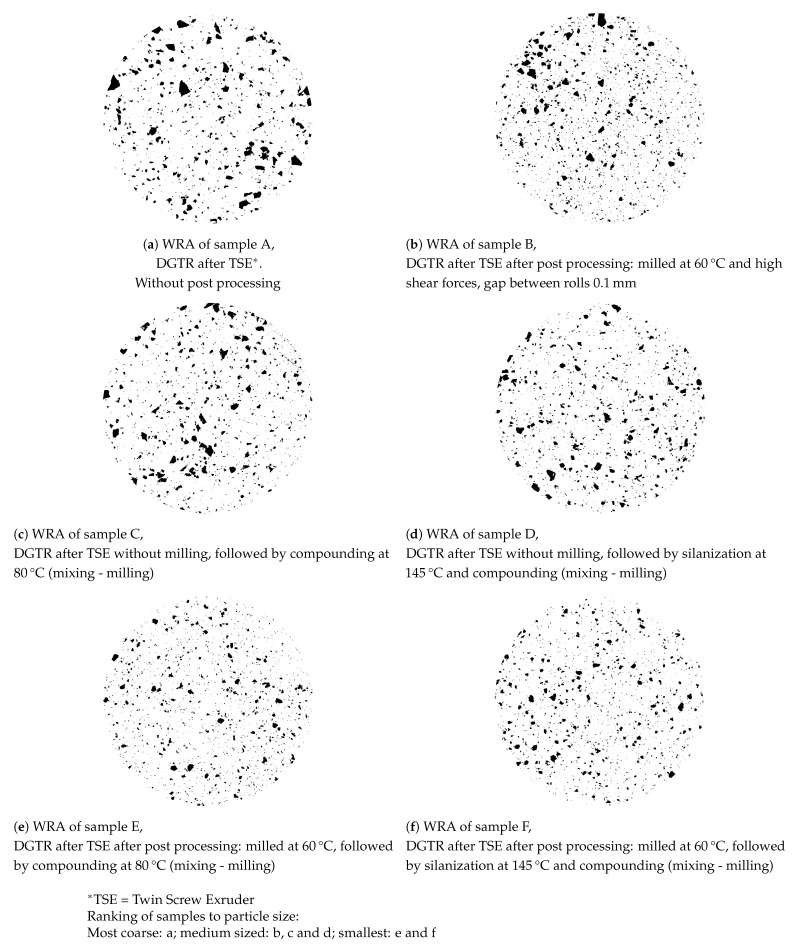
Influence of post processing on size and amount of visible grains in DGTR by WRA. The diameter of the samples is 50 mm.

At low temperature the shear forces increase and break the polymer network to a greater extent, and at the same time the reactivity of the free radicals decreases exponentially. At too low temperatures however, the degree of softening of the de-vulcanizates decreases considerably. At 60 °C a workable balance was found. An advantage of post-process milling as an addition to the de-vulcanization process in the extruder is that the de-vulcanizate gains coherency in spite of the still remaining visible grains. A clear difference observed during milling concerns the cohesive elastic nature of the de-vulcanized GTR compared to the granular consistency of the only cooled de-vulcanizate from the extruder. This implies that the shear forces during milling do not destroy the rubber matrix, but only aid in shearing off the layers of de-vulcanized material. This is shown by comparing the size of the remaining visible grains after different treatments by WRA as shown in Figure 13:

- The devulcanizate, produced by the extruder and directly cooled by the cooling calender, clearly contains remaining grains, as is shown by WRA of sample A.
- These reduce in size by additional milling at 60 °C, post-proces milling, as is shown by WRA of sample B
- When the devulcanizate of sample A is compounded in an internal mixer at 80 °C, as described in Table A3 or at 145 °C, as described in Table A4, see the WRA of samples C and D, the size of the visible grains is about similar compared to the WRA of sample B. Hence there is no substantial change in size of the remaining visible grains by the additional processing steps.
- When the devulcanizate of sample A is milled at 60 °C as for sample B, prior to the processing steps as described for the samples C and D, the size of the remaining grains reduces considerably, as is shown by the WRA of samples E and F.
- This clearly shows that an additional milling of the devulcanizate prior to processing for re-vulcanization has a large influence on the reduction in size of the visible grains, while the processing for revulcanization only add a small additional reduction in size of the visible grains. There is only a minor effect noticeable of the temperature or the mixing process of the preparation for re-vulcanization on the size of the remaining visible grains.

This is indicated in Table 6.

As derived in the theoretical Section 2, a gradient in concentration of the de-vulcanization agent in the GTR-particles caused by differences in size distribution of GTR can be expected due to migration limitations. Consequently, a gradient in decreased cross-link density in the particles can be expected. This also implies a gradient in hardness of these particles and thus a decreasing level of “erosion” [24] the de-vulcanized layers when applying high shear forces by post-process milling. Another advantage of post-process milling is that the de-vulcanizates gain coherency, in spite of the still remaining visible grains. Ahagon and Kirino [5] indicated that large deformations in SBR rubber on a mill can decrease the cross-link density at first, while increasing it later on. However, this is not observed in the present process during post-process milling of the devulcanized GTR.

### 4.7. Concluding Remarks for Future Improvement Steps

The set-up presented in this study deserves further improvement in terms of higher production capacities. The following suggestions can be made:

- For thorough mixing of GTR with the de-vulcanization agent, melting and allowing adequate time for migration of the DBD into the granulate particles at a temperature of 130 °C, a separate mixer step prior to de-vulcanization in the extruder is appropriate.
- The optimal conditions for the de-vulcanization process are a residence time of about 12 min at a de-vulcanization temperature of 180 °C and only a limited amount of mixing: A twin screw extruder is in principle suitable, although the relatively long residence time needed for the de-vulcanization limits the capacity. For the present set up this was partly solved by use of the elongated die.
- Applying high shear at a temperature of 60 °C by post-process milling on a simple laboratory mill for about 5 min with a gap of 0.1 mm between the rolls improved the structure and properties of the de-vulcanizates. As this is not a practical solution for a continuous process, preliminary experiments with a single screw extruder using the cooled de-vulcanizate after the twin screw extruder showed promising results: such a set up can be easier implemented instead of the discontinuous milling process.

The outcome of the present study provides a valuable contribution to solve a major environmental problem: the need for high quality recycled passenger car tire rubber containing silica as reinforcing filler.

## 5. Conclusions

This study described the adaptations needed for screw configuration and settings of a twin-screw co-rotating extruder, as dictated by the change from DBDS to DBD as de-vulcanization agent, as well as additional optimizations for the de-vulcanization of passenger car tire rubber.

A crucial factor was the occurrence of silica in the tire rubber used as compounding ingredient in GTR of recent date. This raised substantial problems due to the fact that this material resulted in a different crosslink structure than a carbon black reinforced rubber:
The occurrence of remaining visible grains in WRA of the de-vulcanizates had a direct relation with the ratio between size of the smallest and largest visible grains in the GTR and the absolute size of the hereof,
It was shown that thermo-chemical-mechanical de-vulcanization can be performed in two steps: a mainly thermo-chemical process at the de-vulcanization temperature in the twin-screw extruder, and the mechanical part of the process at a much lower temperature on a mill. In this way the extruder process was optimized concerning tensile properties, while at the same time the morphology of the de-vulcanizates was improved and the size of the remaining visible grains was reduced.
The Horikx-Verbruggen analysis method, in principle a criterion of the quality of the de-vulcanizates, should be used with care for GTR comprised of a blend of both carbon black and silica-silane fillers.
The sample with the best tensile strength after re-vulcanization was obtained with 4 wt% de-vulcanization agent DBD, 2 wt% process oil and 1 wt% TDTBP temperature of the feed- and mixing-section of the twin-screw extruder set at 130 °C, the temperature of the de-vulcanization section set at 180 °C and the temperature of the pressure section at 150 °C; screw configuration D, as shown in Figure 6, and operated at a speed of 20 rpm, to obtain a residence time of 12 min.
For re-vulcanization. The best vulcanization system within this study turned out to be 4.6 phr sulfur, 4.6 phr TBBS, 3.2 phr TESPT and 2.8 phr DPG, including silanization. A best tensile strength of 8 MPa at a strain at break of 160% of the unblended re-vulcanizate was found.
The capacity of the twin-screw extruder was severely limited by the required residence time, implying a low screw speed. Part of the available screw length had to be used for mixing the de-vulcanization agent DBD with GTR, melting and allowing time for migration of the DBD into the GTR, before the actual de-vulcanization started. This remains a subject of future optimization.

## Figures and Tables

**Table 1:** Diffusion coefficients D and calculated diffusion times until equilibrium vs. temperature for an oil/Styrene-Butadiene Rubber (SBR) system according to Bouvier and Gelus [16].

Temperature	D	Time	
[°C]	[μm${}^{2}$/s]	d = 2 mm	d = 3 mm
20	16	17 h	39 h
100	556	30 min	67 min
163	1690	10 min	22 min
180	2530	7 min	15 min
201	3390	5 min	11 min

**Table 2:** Size distribution of the GTR after analysis with laboratory sieves.

Size Sieve Mesh [mm${}^{2}$]	Amount [wt%]
<0.85	negligible
0.85 < GTR < 2	80
2 < GTR < 3.5	19
>3.5	1

**Table 3:** Tensile values after re-vulcanization with a silica-silane based formulation. Values after de-vulcanization using screw A, revulcanized with formulation II, Table A3.

Sample	DBD	Temperature Setting	Screw	TDAE	Tensile		Strain		M100	
		for Sections I-II-III ${}^{1}$	Speed		Stress	std ${}^{2}$	at Break	std ${}^{2}$		std ${}^{2}$
	wt%	°C	rpm	wt%	MPa	MPa	%	%	MPa	MPa
aA2	5	130-220-150	10	2	7.6	0.4	137	3	4.4	0.1
bA2	3.9	100-220-100	10	6.2	7.2	0.5	139	7	4.3	0.1
cA2	6.85	100-220-220	10	6.2	6.9	0.7	179	12	4.8	0.1
dA2	6.85	100-220-100	10	6.2	6.1	0.5	134	8	3.9	0.1
eA2	5	130-220-150	17	2	6.1	0.6	119	9	3.1	0.2
fA2	5	130-220-120	10	5	5.9	0.6	127	7	4.3	0.1
gA2	6.85	100-220-100	10	6.2	5.8	0.6	142	8	5.1	0.1
hA2	5	130-220-120	17	5	5.5	0.2	125	5	4.5	0.2
iA2	6.85	220-220-220	34	6.2	5.3	0.5	121	6	4.1	0.1
jA2	6.85	220-220-220	34	6.2	5.2	0.4	121	7	3.9	0.1
kA2	6.85	220-220-220	34	6.2	5.2	0.3	121	6	4.1	0.1
lA2	3.9	100-220-100	10	6.2	5.1	0.8	118	13	4.7	0.1
mA2	6.85	220-220-220	10	6.2	4.9	0.5	119	8	3.9	0.1
nA2	6.85	220-220-220	10	6.2	4.6	0.7	107	13	4	0.1
oA2	6.85	220-220-220	10	6.2	4.5	0.5	109	7	4.1	0.1

${}^{1}$: I = Feed& mixing section, II = de-vulcanization section, III = Pressure section, see Figure 2, ${}^{2}$: Standard deviation over 5 samples.

**Table 4:** Tensile values after re-vulcanization with a silica-silane based formulation. Values after de-vulcanization using screw D, revulcanized with formulation I, Table A3.

Sample	DBD	Temperature Setting	Screw	TDAE	Tensile		Strain		M100	
		for Sections I-II-III ${}^{1}$	Speed		Stress	std ${}^{2}$	at Break	std ${}^{2}$		std ${}^{2}$
	wt%	°C	rpm	wt%	MPa	MPa	%	%	MPa	MPa
aD1	6.85	130-180-170	20	2	8	0.06	176	111	3.8	0.1
bD1	3.9	130-220-150	20	2	7.6	0.4	131	5	5.2	0.1
cD1	6.85	130-220-150	20	2	7.5	1.1	140	15	4.7	0.1

${}^{1}$: I = Feed& mixing section, II = de-vulcanization section, III = Pressure section, see Figure 2, ${}^{2}$: Standard deviation over 5 samples.

**Table 5:** Tensile values after re-vulcanization with a silica-silane based formulation. Values after de-vulcanization using screw D, revulcanized with formulation II, Table A3.

Sample	DBD	Temperature Setting	Screw	TDAE	Tensile		Strain		M100	
		for Sections I-II-III ${}^{1}$	Speed		Stress	std ${}^{2}$	at Break	std ${}^{2}$		std ${}^{2}$
	wt%	°C	rpm	wt%	MPa	MPa	%	%	MPa	MPa
aD2	3.9	130-180-170	20	2	8.2	0.4	158	6	4.4	0.1
bD2	6.85	130-220-150	30	2	7.8	0.3	161	4	4	0.1
cD2	6.85	130-220-150	20	2	7.8	0.4	156	4	4.4	0.1
dD2	3.9	130-220-150	20	2	7.6	0.5	136	7	3.5	0.1
eD2	6.85	130-220-150	20	2	7.3	0.2	147	3	4.6	0.1
fD2	6.85	130-220-150	20	5	7.1	0.3	153	4	4.7	0.1
gD2	3.9	130-220-150	30	2	7.1	0.5	137	5	4	0.1
hD2	6.85	130-180-170	20	2	6.9	0.3	141	7	4.3	0.2
iD2	6.85	130-220-110	10	5	6.7	0.7	133	9	4.1	0.1
jD2	6.85	130-220-170	20	2	6.6	0.4	145	6	4.1	0.1
kD2	5	130-220-150	20	2	6.6	0.4	131	6	3.9	0.1
lD2	3.9	130-220-150	20	2	6.5	0.7	134	9	4.6	0.1
mD2	6.85	130-220-170	34	5	6.4	0.4	156	6	5	0.1
nD2	6.85	130-220-150	20	2	6.4	0.5	139	6	4.1	0.1
oD2	6.85	130-220-130	10	5	6.4	0.3	133	5	4.2	0.1
pD2	5	130-180-170	30	2	6.3	0.5	118	7	3.6	0.1
qD2	6.85	130-220-150	10	5	5.9	0.3	133	4	4.7	0.1
rD2	6.85	130-220-100	10	2	5.9	0.6	125	9	4.9	0.1
sD2	3.9	130-180-170	20	2	5.8	0.4	119	8	4.1	0.1

${}^{1}$: I = Feed& mixing section, II = de-vulcanization section, III = Pressure section, see Figure 2, ${}^{2}$: Standard deviation over 5 samples.

**Table 6:** Summary of investigated processing steps after de-vulcanization with respect to the influence on particle size as indicated by WRA, as shown in Figure 13.

Processing Steps	Sample for WRA					
	A	B	C	D	E	F
De-vulcanizate as produced by the twin screw extruder (TSE) and cooled by calendering,	*	*	*	*	*	*
Additionally milled at 60 °C, with a gap between the rolls of 0.1 mm and a friction ratio of 1.13,		*			*	*
Compounded for re-vulcanization at 80 °C according to Formulation I, Table A2, including sheeting off on the mill and not revulcanized yet,			*		*	
Compounded for re-vulcanization at 145 °C according to Formulation II, Table A4, including sheeting off on the mill and not revulcanized yet.				*		*
Relative size and number of remaining visible grains (less ∘∘∘ is smaller and/or less)	∘∘∘∘	∘∘∘	∘∘∘	∘∘∘	∘	∘∘
Reference to	Figure 13a	Figure 13b	Figure 13c	Figure 13d	Figure 13e	Figure 13f

## Data Availability

The data presented in this study are available on request from the corresponding author.

## Abbreviations

The following abbreviations are used in this manuscript:

BR	Butadiene Rubber
B.V.	Besloten Vennootschap, similar to Ltd or Inc.
DBD	2-2${}^{\prime }$-dibenzamidodiphenyldisulfide, peptizer, de-vulcanization agent
DPDS	diphenyl disulfide, de-vulcanization aid
DGTR	Devulcanized granulated passenger car tire rubber
DPG	1,3-diphenylguanidine
EPDM	Ethylene-propylene-diene terpolymer
GTR	Granulated passenger car tire Rubber
NR	Natural rubber
IIR	Butyl rubber
MBTS	Mercapto benzotiazoledisulfide, curative
phr	Per hundred rubber, equivalent with per hundred polymer
SBR	Styrene-butadiene rubber
TBBS	*N*-tert-butyl-2-benzothiazolesulfenamide, curative
TDAE	Treated distillate aromatic extract, process oil
TDTBP	Tris(2,4-di-tert-butylphenyl)phosphite, anti-oxidant, stabilizer
TESPT	bis[3-(triethoxysilyl)propyl: tetrasulfide, coupling agent
THF	Tetrahydrofuran, solvent
TMQ	2,2,4-trimethyl-1,2-dihydroquinoline, anti-oxidant
6PPD	*N*-(1,3-dimethylbutyl)-N${}^{\prime }$-phenyl-p-phenylenediamine, anti-ozonant

## Appendix A

### Appendix A.1. Preparation of the Re-Vulcanizates for Tensile Testing Based on CB as Active Filler in GTR

For the tensile strength analysis, the de-vulcanizates were re-vulcanized with the formulation in Table A1. This is based on a tire tread formulation under the assumption that the de-vulcanizate is solely carbon black reinforced. The corresponding compounding procedure is described in Table A2.

**Table A1:** Re-vulcanization formulation for a de-vulcanized carbon black based tire tread, values in parts per hundred rubber (phr).

Component	
Polymer (1)[x]	[100]
ZnO	3.0
St.A.	2.0
TDAE$\left[x\right]$	[42.7]
Carbon black(2)$\left[x\right]$	[80]
6PPD	1.0
TMQ	2.0
TBBS	1.5
Sulphur	1.5

$\left[x\right]$ Components already present in de-vulcanizate. (1) Total polymer content of (D)GTR, a mix of mainly SBR, BR and NR. (2) All fillers treated as if they were carbon black.

**Table A2:** Compounding procedure for the carbon black based re-vulcanization formulation as given in Table A1.

	**Mixer Settings**
	Brabender internal mixer
	Chamber volume: 35 mL
	Fill factor: 0.6
	Initial mixer temperature of 80 °C.
	Mixer set at 5 rpm rotor speed.
**Time [minutes]**	**Processing Steps**
	Addition of curatives.
	Mixing order:
0	Mixer set at 50 rpm,
1	ZnO + stearic acid,
4	TBBS
4.5	Sulphur
5	Dump
	Thoroughly homogenized and sheeted off on the mill.
	Relaxation for 24 h.

### Appendix A.2. Preparation of the Re-Vulcanizates for Tensile Testing Based on Both CB and Silica as Active Fillers in GTR

Taking the presence of silica as reinforcing filler also into account, the curing system had to be adjusted as shown in Table A3, Formulation I. Formulation II, with an additional amount of sulfur and TBBS, was chosen as it gave more consistent tensile results [14]. The corresponding compounding procedure is given in Table A4. A relaxation time of 72 h was used after the silanization stage, as the re-vulcanizates showed better tensile properties.

**Table A3:** Re-vulcanization formulations for a silica and carbon black containing de-vulcanizate, derived from a silica based tread formulation, values in phr.

	Formulation	
Component:	I	II
Polymer (1)[x]	[100]	[100]
ZnO	2.5	2.5
St.A.	1.0	1.0
TDAE$\left[x\right]$	[42.7]	[42.7]
Carbon black$\left[x\right]$	[54] (3)	[54] (3)
Silica$\left[x\right]$	[42] (3)	[42] (3)
TESPT	3.2	3.2
TBBS	1.64	4.64
DPG	2.8	2.8
Sulphur	1.64	4.64

$\left[x\right]$ Components already present in de-vulcanizate. (1) Total polymer content of (D)GTR, a mix of mainly SBR, BR and NR. (2) Additional sulfur and TBBS added because of increased repeatability of tensile measurements. (3) Based on experiments concerning the composition of GTR.

**Table A4:** Two stage compounding procedure for silica/carbon black based formulations as given in Table A3.

**Time [Minutes]**	**Mixer Settings and Processing Steps for STAGE 1**
	Brabender internal mixer
	Chamber volume: 35 mL
	Fill factor 0.6
	The mixer temperature was set to 145 °C.
	The de-vulcanizate was added at 5 rpm rotor speed.
	Silanization.
0	Mixer set at 50 rpm rotor speed.
1	Silane.
5	Dump at approximate 145 °C.
0	The de-vulcanizate was cooled down to 60 °C and milled with a gap-width of 0.1 mm to 0.5 mm.
5	Sheeted off at 2 mm.
	Relaxation for 72 h.
**Time [Minutes]**	**Mixer Settings and Processing Steps for Stage 2**
	Initial mixer temperature of 50 °C and 5 rpm rotor speed.
	The silanized de-vulcanizate was added at 5 rpm rotor speed.
	Addition of curatives.
0	Mixer set at 50 rpm rotor speed.
0.5	ZnO + stearic acid.
1	TBBS + DPG.
1.5	Sulphur.
2	Dump.
	Thoroughly homogenized and sheeted off on the mill.
	Relaxation for 24 h.

## References

[1] Musacchi E. Recycled Tyre Materials. Proceedings of the 4th Annual International Recycled Rubber Products (R2P) Technology Conference.

[2] Sienkiewicz M., Kucinska-Lipka J., Janik H., Balas A. (2012). Progress in used tyres management in the European Union: A review. Waste Manag..

[3] Institute of Scrap Recycling Industries Inc. (2016). Factsheet Tires USA. Rubber Chem. Technol..

[4] Golub M.A. (1978). Thermal Rearrangements of Unsaturated Polymers. Rubber Chem. Technol..

[5] Ahagon A., Kirino Y. (2007). Mechanochemical Reactions in Black-Filled SBR Vulcanizates under Large Deformation. Rubber Chem. Technol..

[6] Huntink N.M., Datta R.N., Noordermeer J.W.M. (2004). Addressing Durability of Rubber Compounds. Rubber Chem. Technol..

[7] Rajan V.V., Dierkes W.K., Noordermeer J.W.M., Joseph R. (2005). Model compound studies on the de-vulcanization of natural rubber using 2,3-dimethyl-2-butene. Rubber Chem. Technol..

[8] Markl E., Lackner M. (2020). Devulcanization technologies for recycling of tire-derived rubber: A review. Materials.

[9] Saiwari S., Dierkes W.K., Noordermeer J.W.M. (2013). de-vulcanization of Whole Passenger Care Tire Material. Kautsch. Gummi Kunststoffe.

[10] Sutanto P. (2006). Development of a Continuous Process for EPDM De-Vulcanization in an Extruder. Ph.D. Thesis.

[11] Saiwari S. (2013). Post-consumer tires back into new tires. Ph.D. Thesis.

[12] Saiwari S., van Hoek J.W., Dierkes W.K., Reuvekamp L.E.A.M., Heideman G., Blume A., Noordermeer J.W.M. (2016). Upscaling of a batch de-vulcanization process for ground car tire rubber to a continuous process in a twin-screw extruder. Materials.

[13] Verbruggen M.A.L., van der Does L., Dierkes W.K., Noordermeer J.W.M. (2016). Experimental validation of the Charlesby and Horikx model applied to de-vulcanization of sulfur- and peroxide-cured vulcanizates of NR and EPDM. Rubber Chem. Technol..

[14] van Hoek J.W., Heideman G., Noordermeer J.W.M., Dierkes W.K., Blume A. (2019). Implications of the use of silica as active filler in passenger car tire compounds on their recycling options. Materials.

[15] Bird R.E., Stewart W.E., Lightfoot E.N. (1960). Chapter 11, Temperature Distributions with More than One Independent Variable. Transport Phenomena.

[16] Bouvier J.M., Gelus M. (1986). Diffusion of Heavy Oil in a Swelling Elastomer. Rubber Chem. Technol..

[17] (2016). Thermal Properties of Polymers. https://www.netzsch-thermal-academy.com/en/.

[18] (2014). Technical Data Sheet Rubber Granulate. https://www.genan.eu/wp-content/uploads/2020/11/2020_2_tds_genan-medium-granulate_uk.pdf.

[19] Horikx M.M. (1956). Chain scissions in a polymer network. Rubber Chem. Technol..

[20] Flory P.J., Rehner J. (1943). Statistical Mechanics of Cross-Linked Polymer Networks in Rubberlike Elasticity. J. Chem. Phys..

[21] Kraus G. (1963). Swelling of Filler-Reinforced Vulcanizates. J. Appl. Polym. Sci..

[22] Porter M. (1967). Structural Characterization of Filled Vulcanizates Part 1. Determination of the Concentration of Chemical Crosslinks in Natural Rubber Vulcanizates Containing High Abrasion Furnace Black. Rubber Chem. Technol..

[23] Schelling P.B. (2015). Rubber Recycling Calender. Bachelor’s Thesis.

[24] Donald L., Manas-Zloczower I.F. (2009). Dispersive Mixing of Solid Additives. Mixing and Compounding of Polymers Theory Practice.

